# Analysis of β-tubulin-carbendazim interaction reveals that binding site for MBC fungicides does not include residues involved in fungicide resistance

**DOI:** 10.1038/s41598-018-25336-5

**Published:** 2018-05-08

**Authors:** David Vela-Corcía, Diego Romero, Antonio de Vicente, Alejandro Pérez-García

**Affiliations:** Instituto de Hortofruticultura Subtropical y Mediterránea “La Mayora”, Universidad de Málaga-Consejo Superior de Investigaciones Científicas (IHSM-UMA-CSIC), Departamento de Microbiología, Universidad de Málaga, Bulevar Louis Pasteur 31 (Campus Universitario de teatinos), 29071 Málaga, Spain

## Abstract

Methyl benzimidazole carbamate (MBC) fungicides are fungicidal compounds that exert their biological activities by preventing cell division through the inhibition of tubulin polymerization, which is the major component of microtubules. Several mutations in the β-tubulin gene contribute to MBC resistance, the most common and significant of which occur at residues 198 and 200. Despite nearly 50 years of agricultural use, the binding site of MBCs and the precise mechanism by which those mutations affect fungicide efficacy have not been determined. The aim of this work was to clarify the mode of action and the mechanism of resistance to MBC fungicides in *Podosphaera xanthii*, the primary causal agent of cucurbit powdery mildew, using a combination of biochemical, biophysical and computational approaches. The results allow us to propose an MBC binding site in β-tubulin that lies close to the GTP binding site and does not include residue 198 involved in MBC resistance.

## Introduction

Methyl benzimidazole carbamate (MBC) fungicides were introduced in the market in the early 1970s and became widely used in agriculture. MBCs are potent inhibitors of tubulin polymerization and exert their antifungal activities by targeting the β-tubulin subunit of the microtubules, which results in the arrest of microtubule formation and a failure in cell division, subsequently leading to cell death^1^. The active moiety in these compounds is methyl-2-benzimidazole carbamate (MBC), also known as carbendazim, from which the classification as MBC fungicides originates^2^. Commercial MBCs currently available include benomyl, carbendazim, thiabendazole and thiophanate-methyl^2^. MBC fungicides are registered in numerous countries worldwide for many crops, including cereals, grapes, fruits, vegetables and others. MBCs control a remarkably broad spectrum of plant pathogenic fungi but do not control oomycetes^3,4^.

MBC fungicides also represent the beginning of serious problems of pathogen resistance to fungicides. A few years following their introduction into the market, loss of disease control with MBC fungicides was reported for several crops, particularly with pathogens that have numerous cycles per year, such as grey mould. Since commercialization, at least 100 species of plant pathogenic fungi have developed a certain degree of resistance to MBCs (www.frac.info). Several mutations in the β- tubulin gene have been associated with resistance to MBC fungicides in phytopathogenic fungi^5^. The most common and significant mutations occur at positions 198 and 200; in particular, the substitution of glutamic acid with alanine, valine or glycine at position 198 and the substitution of phenylalanine with tyrosine at position 200 confer high and intermediate levels of resistance to MBC fungicides, respectively^6–8^. Powdery mildew fungi (*Erysiphales*) are a particular group of plant pathogens that annually threaten a variety of crops worldwide, causing economically important losses. Numerous vegetable crops are susceptible to powdery mildew fungi; however, cucurbits are arguably the most severely affected^9^. *Podosphaera xanthii* is considered the main etiological agent of powdery mildew on cucurbits and is one of the most important limiting factors for cucurbit production^10,11^. The management of *P. xanthii* relies on the intensive application of fungicides, and MBCs occupied a relevant position when they were released, however, resistant isolates began to appear a few years after its introduction in field for disease control, reducing the effectiveness of this fungicides family^12^.

We have previously demonstrated that the substitution of glutamic acid with alanine at position 198 (E198A) is responsible for MBC resistance in *P. xanthii*^13^. After more than 40 years of agricultural use, an experimentally determined crystal structure of a fungicide-β-tubulin complex remains unavailable and the precise mechanism of MBC binding remains unknown. Moreover, to date, no studies have reported the molecular mechanism by which mutations in the β-tubulin gene prevent fungicide binding. In this study, we aimed to investigate the mode of action and the mechanism of resistance to MBC fungicides using *P. xanthii* tubulins as a model system to analyze fungicide-protein interactions. Using a combination of biochemical, biophysical and computational approaches, we provide new insights that allow us to propose an MBC binding site in β-tubulin that does not include residues involved in fungal resistance. We also demonstrate that the E198A mutation slightly alters the structure of the β-tubulin, which enables resistance to the fungicide.

## Results

### The E198A mutation in β-tubulin confers resistance to carbendazim in *Podosphaera xanthii* and *Schizosaccharomyces pombe*

**Figure 1:**
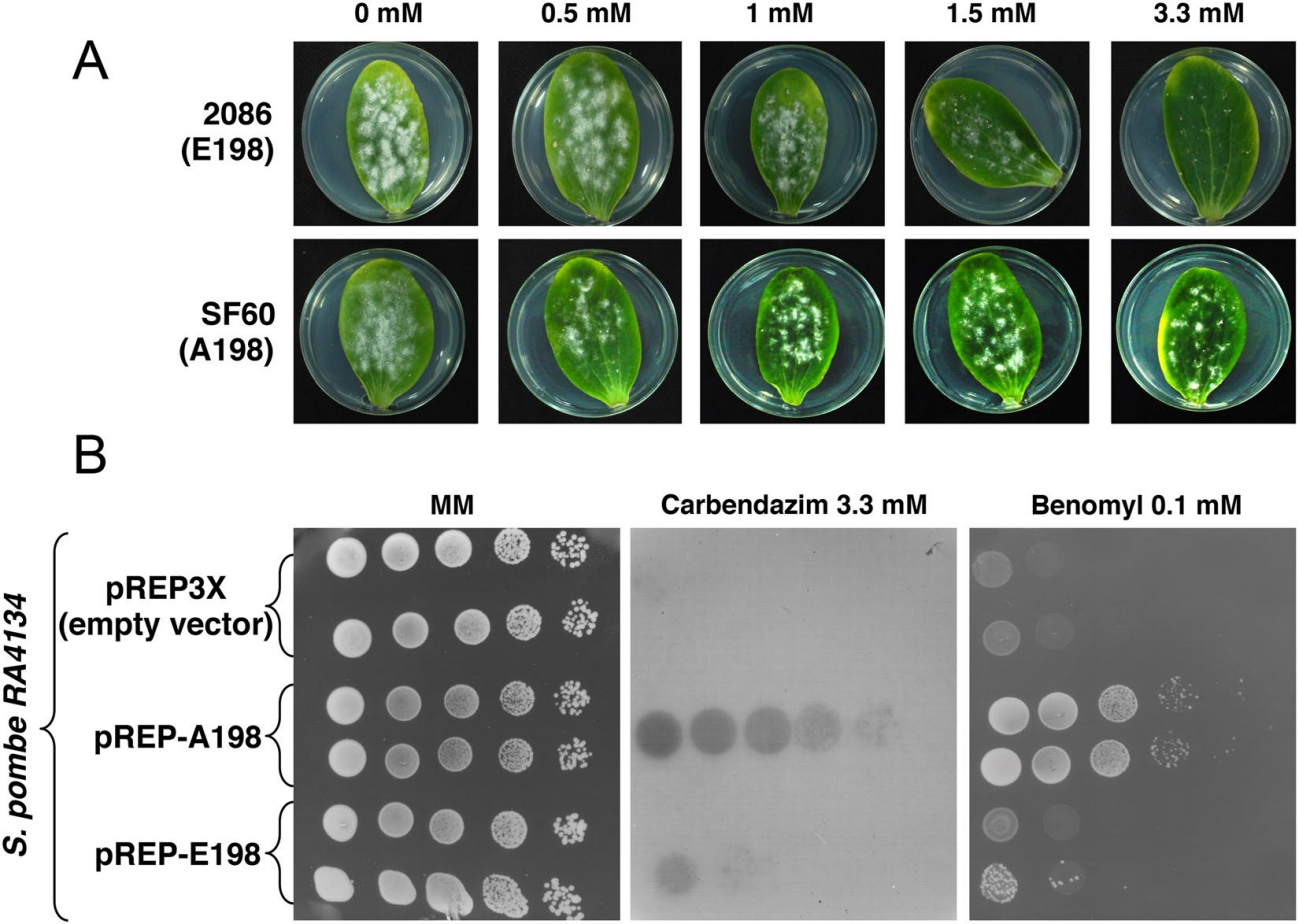
The E198A mutation in the *P. xanthii* β-tubulin confers resistance to the MBC fungicide carbendazim. (**A**) The *P. xanthii* SF60 (A198) isolate grew on zucchini cotyledons treated with increasing concentrations of carbendazim until 3.3 mM. However, the growth of isolate 2086 (E198) was completely abolished at 1.5 mM. (**B**) Coding sequences of the *P. xanthii TUB*2 gene were cloned in the yeast expression vector pREP-3X and plasmids were then transformed into *S. pombe* strain RA4134. The sensitivity of *S. pombe* transformants to fungicides was evaluated by drop assay onto PMG medium lacking thiamine, without fungicide or amended with the MBC fungicides carbendazim (3.3 mM) or benomyl (0.1 mM). The strain expressing the resistant β-tubulin (pREP-A198), but not the strain expressing the sensitive β-tubulin (pREP-E198), grew in the presence of 3.3 mM carbendazim. Identical results were obtained using the MBC fungicide benomyl (0.1 mM).

We have previously identified a point mutation that resulted in an amino acid substitution at position 198 from glutamic acid to alanine in the β-tubulin subunit that correlated with resistance to MBC fungicides in isolates of *P. xanthii*^13^. We initially sought to evaluate the effect of increasing concentrations of carbendazim on growth of a strain (isolate SF60) bearing the MBC-resistant allele (A198) and of a strain (isolate 2086) bearing the MBC-sensitive allele (E198). As expected, the isolate SF60 was resistant to carbendazim up to concentration of 3.3 mM, which is the maximum concentration authorized for use on crops. However, the isolate 2086 exhibited a progressive inability to grow with increasing concentrations of carbendazim and growth was completely abolished at a concentration of 3.3 mM (Fig. 1A).

If the E198A mutation in β-tubulin was responsible for the carbendazim-resistant phenotype of the natural SF60 isolate, we could tentatively reproduce this phenotype by transferring the mutant A198 allele to another organism that has not been exposed to carbendazim. To evaluate this hypothesis, we transformed the sensitive and the resistant alleles of the *P. xanthii* β-tubulin gene (also known as *TUB2*, since *TUB1* is α-tubulin) into the fission yeast *S. pombe*, and the transformants were assayed for resistance to carbendazim, using 3.3 mM as a discriminatory concentration. As anticipated, the *S. pombe* strain RA4134 expressing the mutant A198 allele grew on PMG medium supplemented with 3.3 mM of carbendazim, whereas the expression of the sensitive E198 *TUB*2 did not support the growth of *S. pombe* at this carbendazim concentration (Fig. 1B). Because the high concentration of carbendazim obstructed the adequate visualization of yeast growth, we included benomyl, which also belongs to the MBC family, at a concentration of 0.1 mM, a concentration previously found to discriminate between resistant and sensitive strains of *Saccharomyces cerevisiae*^14,15^. As previously observed for carbendazim, the fission yeast strain carrying the resistant allele but not the strain carrying the sensitive allele was resistant to benomyl (Fig. 1B). Thus, we confirmed that the E198A mutation found in the β-tubulin gene was responsible for the resistance to MBC fungicides observed in *P. xanthii*.

### The E198A mutation induces minor changes in the secondary structure of β-tubulin

Following the determination of the biological significance of the E198A mutation in the β-tubulin subunit, we examined the molecular mechanism that underlies fungal resistance to carbendazim. For this purpose, the α- and β-tubulin genes from *P. xanthii* isolates resistant and sensitive to MBC fungicides were cloned in expression vectors and transformed into *Escherichia coli*, a suitable and successful method for heterologous expression of fungal α- and β-tubulin^16–18^, and also for tubulin-related proteins and other eukaryotic proteins^19–21^. In addition, *E. coli* expression system has been described as a good method for expression of many mammalian proteins^22^. Upon induction with 1 mM IPTG, recombinant α- and β-tubulin were purified to homogeneity and analyzed by SDS-PAGE under reducing conditions (Figure S1A and B). The homogeneity of the protein solutions was further confirmed via immunoblot assays using an anti-histidine specific antibody (Figure S1C) and mass spectrometry analysis (Data S1). We hypothesised that the resistance associated with the E198A mutation may result from an effect on the conformation of the mutant β-tubulin.

Therefore, we first performed circular dichroism (CD) analysis of MBC-sensitive (E198) and MBC-resistant (A198) β-tubulins (Figure S2). The spectrum of the sensitive β-tubulin exhibited two minima at 208 and 222 nm, which are characteristic for the presence of α-helical secondary structure. The CD spectrum of the resistant β-tubulin was not identical to that of wild type β-tubulin. Indeed, the estimation of the percentages of secondary structure for sensitive and resistant β-tubulins using the K2D3 server^23^ indicated a similar α-helical content for both proteins but a slight decrease in the β-sheet content in the resistant β-tubulin (Table S1).

**Figure 2:**
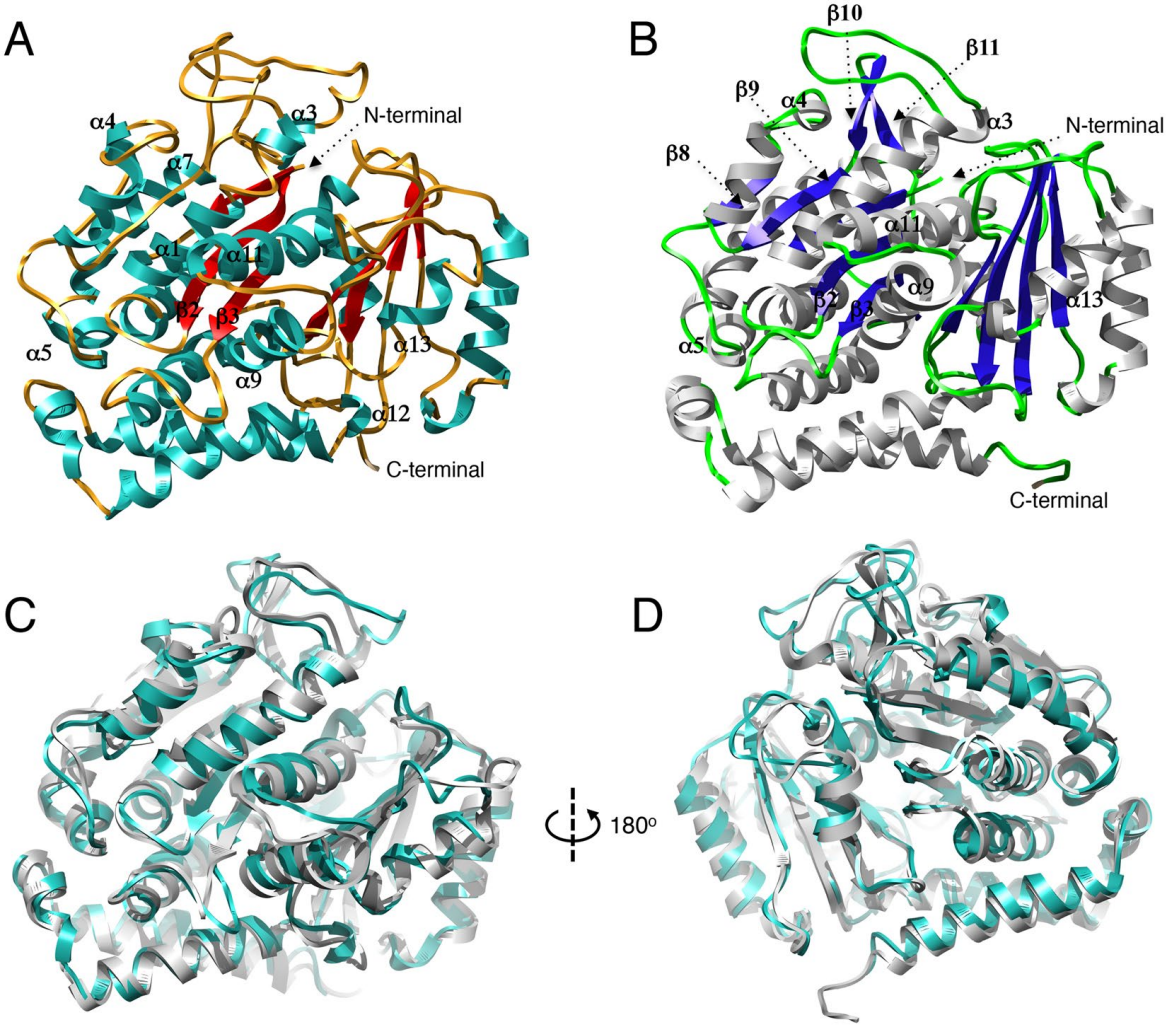
Structural comparison of the three-dimensional models of MBC-sensitive and MBC-resistant *P. xanthii* β-tubulin proteins. Compared to the MBC-sensitive β-tubulin protein (**A**), in the MBC-resistant β-tubulin (**B**) the E198A mutation results in the formation of additional β-strands (β8, β9, β10 and β11). (**C** and **D**) Superposition of MBC-sensitive (cyan) and MBC-resistant (gray) *P. xanthii* β-tubulin models. As observed, both models do not entirely superpose, indicating a conformational change resulting from the E198A substitution. This conformational change is not restricted to a specific protein domain; rather, it triggers a rearrangement of the entire protein structure.

Despite the minor variation observed in the CD spectra, we sought to evaluate a possible global change in the three-dimensional structures of these proteins. We obtained three-dimensional models of sensitive and resistant β-tubulins (Fig. 2A,B) using the previously determined crystal structure of *Bos taurus* β-tubulin (PDB ID: 1JFF) as the template^24^. The QMEAN, a composite scoring function describing the major geometrical aspects of protein structures^25^, of these models was 0.5, supporting the high quality of the models and, thus, their reliability and utility in subsequent analyses. A comparison of both models allows the rapid identification of structural effects associated with the E198A mutation; the structural model of resistant β-tubulin exhibits predominantly β-sheet secondary structure compared with the sensitive variant, with the appearance of new strands (β8, β9, β10 and β11) that are not present in the sensitive model. Additionally, superposition of both models does not result in identical structures (Fig. 2C,D). Thus, the slight variation observed in the secondary structure of the mutant β-tubulin may lead to a rearrangement in the overall structure of the protein (Movie S1), which eventually may confer resistance to carbendazim.

### The E198A mutation prevents the interaction between carbendazim and β-tubulin

**Figure 3:**
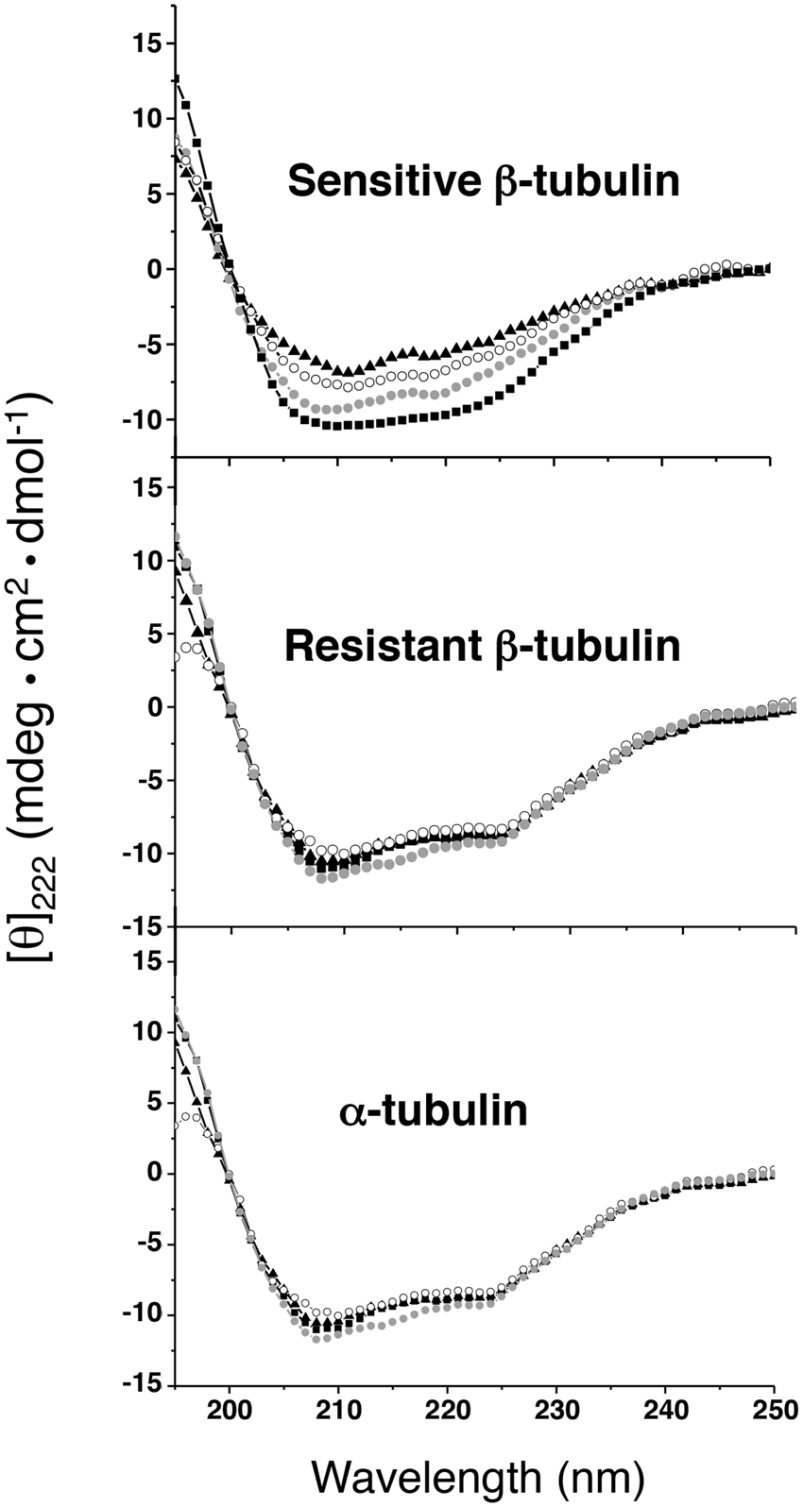
Circular dichroism (CD) analysis of the effect of carbendazim on the secondary structures of *P. xanthii* α-tubulin and β-tubulin. MBC-sensitive and MBC-resistant β-tubulins and α-tubulin were incubated in the absence (■) or presence of 0.5 (), 1.5 (○) and 3.3 mM (▲) carbendazim. Each spectrum represents the average of three scans.

The ability of mutant β-tubulin to confer resistance to MBC fungicides suggested that the specific target in the protein was immune to the fungicide. To explore this hypothesis, we investigated the effect of carbendazim on the secondary structure of β-tubulin proteins using circular dichroism spectroscopy (Fig. 3). Spectra were recorded at room temperature in the absence or presence of various concentrations of carbendazim. The CD spectra of sensitive β-tubulin varied progressively with increasing concentrations of carbendazim; specific changes include the typical minima at 208 and 222 nm, which increased toward positive values. In contrast, the CD spectra of the resistant β-tubulin remained invariable, as observed for α-tubulin, which is not a known target of carbendazim. In addition, circular dichroism analysis of carbendazim alone was performed, as well as a theoretical spectrum from sum in the presence of carbendazim and β-tubulin was represented showing that carbendazim caused no modifications on β-tubulin spectrum (Figure S3).

**Figure 4:**
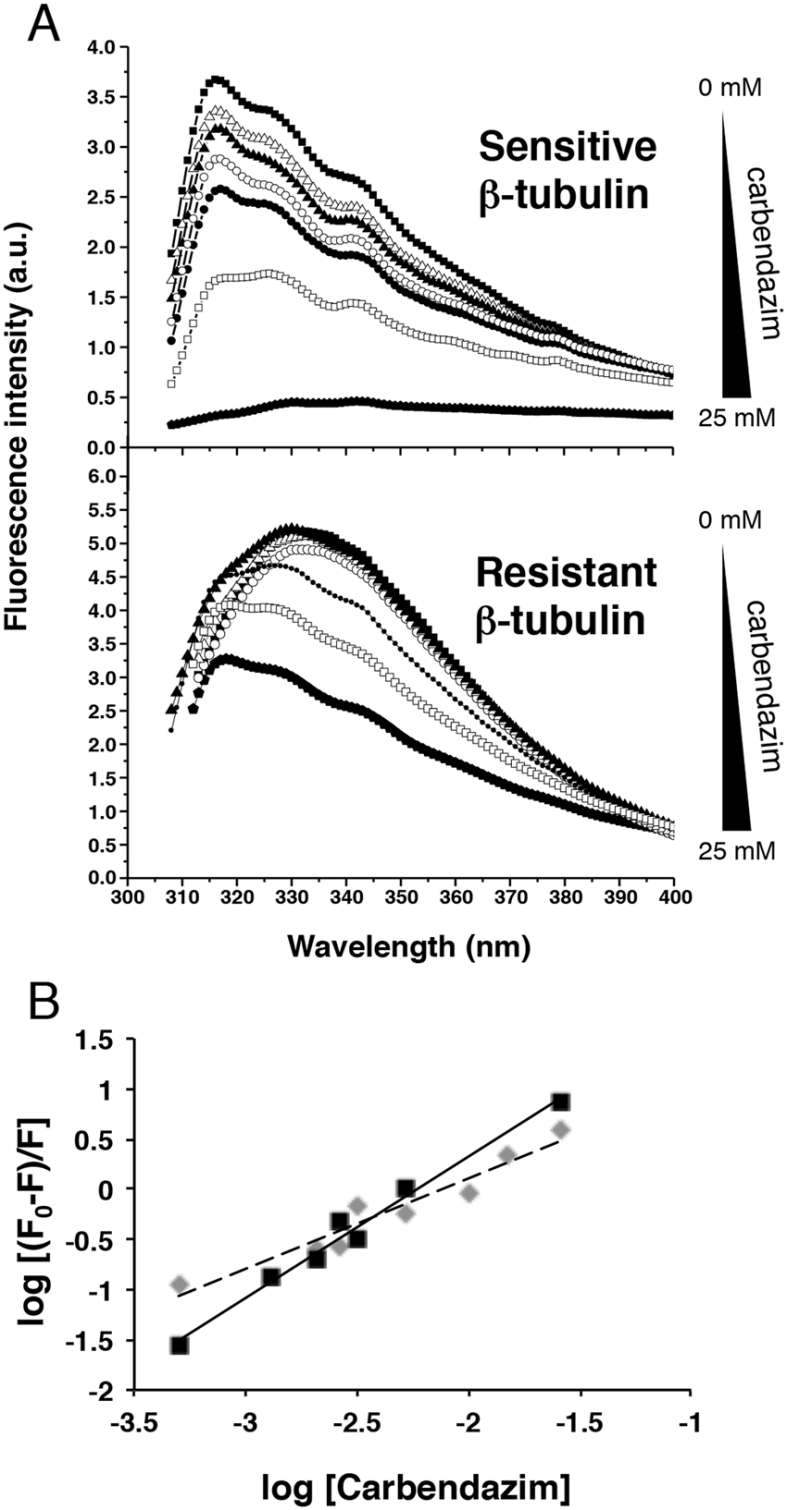
Characterization of the interaction between carbendazim and *P. xanthii* β-tubulins by monitoring the intrinsic tryptophan fluorescence. (**A**) Fluorescence spectra of the MBC-sensitive and MBC-resistant β-tubulins. Sensitive and resistant β-tubulins were incubated in the absence (■) and presence of different concentrations of carbendazim: 1.3 (△), 2.5 (▲), 3.3 (○), 5.2 (●), 15.0 (□) and 25.0 () mM. (**B**) A plot against log [carbendazim concentration] according to the modified Stern-Volmer equation for MBC-sensitive (solid line) or MBC-resistant (dashed line) β-tubulin. *F*_*0*_ and *F* are the peaks of fluorescence intensities of β-tubulin in the absence and presence, respectively, of the quencher (carbendazim).

To emphasize the effect of carbendazim on the secondary structure of β-tubulin, the molar ellipticity at 222 nm ([Θ]_222_) was plotted against the concentration of carbendazim (Figure S4). A reduction in the molar ellipticity of sensitive β-tubulin correlated with increasing carbendazim concentrations. Nevertheless, the resistant β-tubulin did not exhibit this tendency, and similar values were observed in the absence of carbendazim and at the highest carbendazim concentration (Figure S4A). As expected for the non-target α-tubulin, no changes in the molar ellipticity were observed in the evaluated carbendazim concentration range (Figure S4B). In addition, the estimation of the percentages of secondary structure based on the linear dependence between structural fractions and the CD spectra indicated that more α-helices transitioned into β sheets with increasing carbendazim concentrations up to 3.3 mM (Figure S5A). In contrast, resistant β-tubulin exhibited a slight tendency to increase the β-sheet content and decrease the α-helical content only in the presence of the higher concentrations of carbendazim (Figure S5B). The CD analysis supported the notion that carbendazim is less able to interact with the resistant β-tubulin. Thus, we next sought to evaluate this interaction using isothermal titration calorimetry (ITC), which is a highly sensitive method to study ligand-protein interactions. Unfortunately, the insolubility of carbendazim and other MBC fungicides in the buffers required for ITC precluded these measurements (data not shown). We alternatively used fluorescence quenching of the intrinsic tryptophan fluorescence of β-tubulin (Fig. 4A) to characterize the protein-fungicide interaction^26^. Fluorescence emission spectrum of carbendazim was also recorded (Figure S6). The emission maximum of sensitive β-tubulin in the absence of carbendazim was centred at 316 nm. Upon the addition of increasing concentrations of carbendazim up to 3.3 mM, the wavelength of the emission maximum remained nearly unaltered, whereas the fluorescence intensity decreased by 40%. This signature indicates the following: (*i*) the secondary structure of β-tubulin remains nearly unaltered up to a carbendazim concentration of 3.3 mM and (*ii*) initial variations in the tertiary structure are induced. A carbendazim concentration of 5 mM resulted in a slight red shift of 26 nm (from 316 to 342 nm) and strong fluorescence quenching (95%), indicating changes in polarity around the fluorophore of the tryptophan residue. As previously observed in CD analysis, carbendazim did not affect the behavior of the resistant β-tubulin. The emission maximum of the protein was centered at 332 nm in the presence and absence of carbendazim and only slight fluorescence quenching (37%) was observed upon the addition of unusually high concentrations of carbendazim (25 mM).

The strength of this fluorescence quenching analysis lies in the ability to characterize ligand-protein interactions in terms of their thermodynamic parameters. Following fluorescence quenching, the interaction between carbendazim and β-tubulin was analysed using the modified Stern-Volmer equation (Fig. 4B). A comparison of the fluorescence maxima allows the rapid visualization of the different affinities of carbendazim for sensitive and resistant β-tubulin. We observed that the slope of the line formed by the fluorescence maximum of sensitive β-tubulin was higher than that of resistant β-tubulin, thus, indicating a higher affinity of carbendazim for sensitive β-tubulin. Moreover, the binding of carbendazim to β-tubulin occurs in one step because a modification in the slope of the binding plot was not observed throughout the concentration range. Therefore, carbendazim binding to β-tubulin may be an all-or-nothing event. We estimated the magnitude of the Stern-Volmer quenching constant (*K*_*SV*_), the binding constant (*K*) and number of binding sites (*n*) and the *∆G*^0^_binding_ (Table 1). The values of the thermodynamic parameters *K* and *∆G*^0^_binding_ and the quenching constant indicated that carbendazim binding to sensitive β-tubulin was spontaneous and strong. From the value of *n*, we propose that carbendazim binding to sensitive β-tubulin occurs in a single binding site. In contrast, thermodynamic parameters obtained for resistant β-tubulin suggested that although carbendazim may interact with the protein, the number of putative binding sites (*n*) was less than one and the free energy necessary for binding is much larger than that for the sensitive β-tubulin.

**Table 1:** Quenching constants and thermodynamic parameters of the interaction of *P. xanthii* β-tubulin with carbendazim.

	^*a*^*K*sv (M^−1^)	*K*^*b*^ (M^−1^)	n^*c*^	Δ*G*^0^_*binding*_ (kJ mol^−1^)
Sensitive (E198)	285.8	1.38 × 10^3^	1.41	−176.71
Resistant (A198)	148.04	48.2	0.79	−94.73

^a^Stern-Volmer quenching constant.

^b^Binding constant.

^c^Number of binding sites.

### Molecular docking reveals a binding site for MBC fungicides in β-tubulin that does not include residues 198 and 200 involved in fungicide resistance

**Figure 5:**
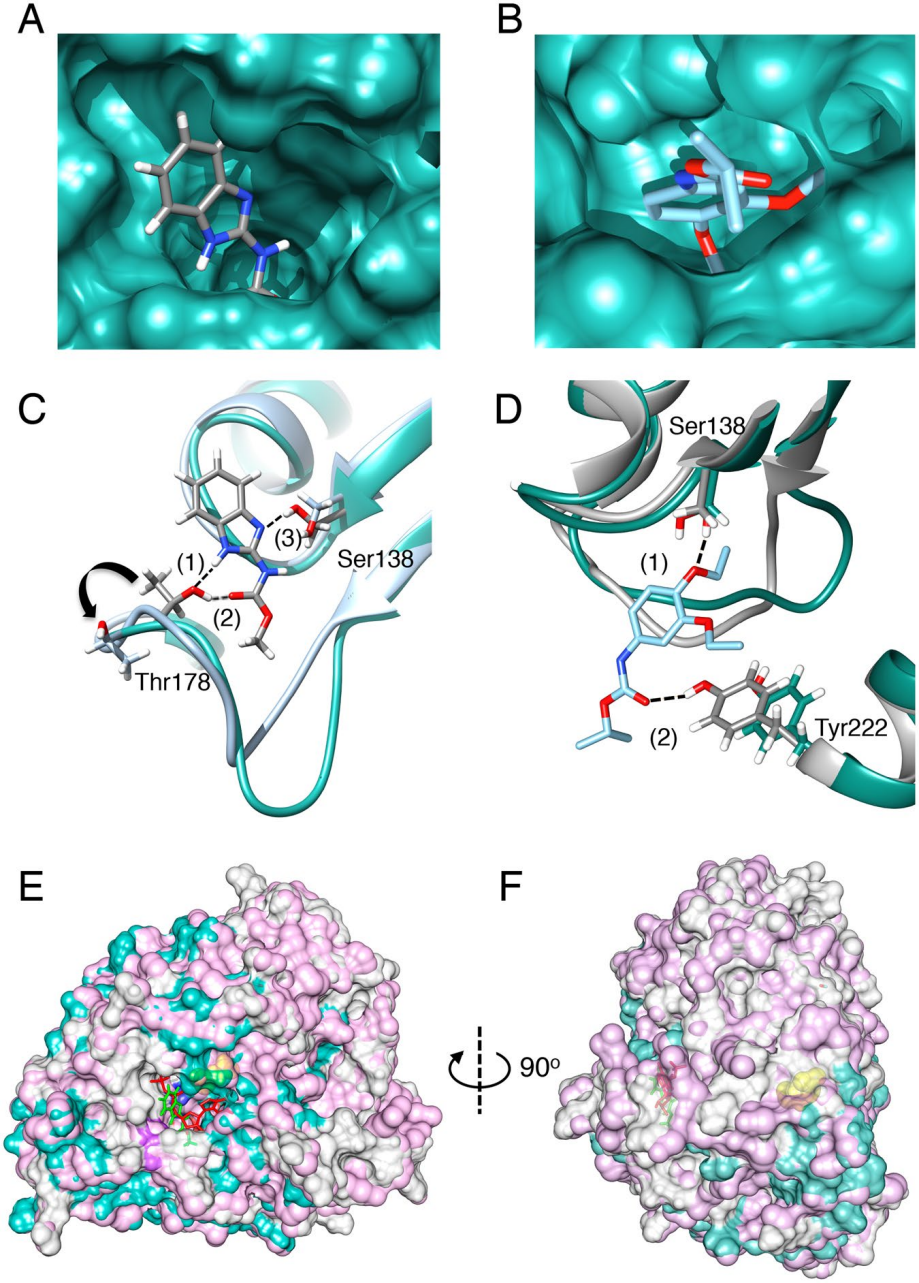
Molecular docking of MBC fungicides to *P. xanthii* β-tubulins. (**A**) Surface model of MBC-sensitive β-tubulin showing the predicted binding site for carbendazim (PubChem ID: 25429) located in the region that binds a new tubulin dimer. (**B**) Surface model of MBC-resistant β-tubulin showing the predicted binding site for diethofencarb (PubChem ID: 91742). (**C**) Docking results of carbendazim combined with a superposition of MBC-sensitive and MBC-resistant β-tubulin models; carbendazim is proposed to bind MBC-sensitive β-tubulin (cyan) *via* three hydrogen bonds (dashed lines) that involve residues Thr178 (1: 2.24 Å; 2: 2.49 Å) and Ser138 (3: 2.1 Å). Thr178 is twisted out (arrow) in the MBC resistant β-tubulin model (grey). (**D**) Docking was also performed using diethofencarb, and it is proposed to bind MBC-resistant β-tubulin (cyan) *via* two hydrogen bonds (dashed lines) that involve residues Ser138 (1: 1.94 Å) and Tyr222 (2: 2.94 Å). By contrast to observe for carbendazim, diethofencarb cannot bind MBC-sensitive β-tubulin (grey) due to the movement of the Tyr222. (**E**) Superposition of a model of mammalian β-tubulin model (cyan; PDB ID: 1JFF) in complex with GTP (red) and the *P. xanthii* β-tubulin model (pink) with carbendazim (green) docked in the proposed MBC fungicide binding site. Alignment results in a high level of coverage between models (grey) and reveals the proximity of the GTP and putative MBC binding sites. Carbendazim binds to β-tubulin *via* residues 138 (blue) and 178 (purple). (**F**) As observed upon 90° rotation of the model, residue 198 (yellow) involved in MBC resistance in *P. xanthii* (the E198A mutation) is located inside of the protein at a position that differs from the proposed MBC binding site).

We performed molecular docking to map the interactions between MBC fungicides and β-tubulin and to identify a putative binding site for these compounds. The molecular docking of carbendazim and sensitive β-tubulin resulted in the identification of 48 clusters in eight different sites of the protein. The top-score cluster exhibited a better “*Full Fitness”*; this parameter is calculated by averaging the 30% most favorable effective energies of a cluster’s element^27^ and lower free energy (−2177.9/−6.36 kcal mol^−1^) than those obtained for other potential binding sites. This putative binding site is formed by residues located in the middle of sensitive β-tubulin (residues 135–142 and 175–185) (Fig. 5A). Molecular docking was also performed using diethofencarb, a N-phenyl carbamate. The model predicted diethofencarb binding in the proposed carbendazim-binding site, however, this case, in resistant β-tubulin (Fig. 5B).

In the interaction with sensitive β-tubulin, carbendazim can form two hydrogen bonds with the hydroxyl groups of Thr178 [(1) 2.24 Å and (2) 2.49 Å] and one with the hydroxyl group of Ser138 (2.1 Å), resulting in a favourable binding site (Fig. 5C). Molecular docking also performed with diethofencarb showed the inability to bind to sensitive β-tubulin at the proposed residues for carbendazim, Thr178 and Ser138, in the orientation observed in sensitive β-tubulin since the distances between residues and the fungicide were not favorable to form hydrogen bonds due to conformational change caused by the substitution E198A. By contrast, diethofencarb was able to bind to resistant β-tubulin by forming one hydrogen bond with the hydroxyl group of Tyr222 (2.48 Å) and one hydrogen bond with the hydroxyl group of Ser138 (1.94 Å), resulting in an encouraging binding to resistant β-tubulin (Fig. 5D). In case of sensitive β-tubulin, hydrogen bond with the hydroxyl group of Tyr222 would be impossible since the distance between this residue and diethofencarb is 6.30 Å. However, due to the topological changes imposed by the substitution E198A, this residue was reoriented in resistant β-tubulin making, then, a more favorable environment for diethofencarb to bind to resistant β-tubulin. This substitution also reoriented the residue Ser138 allowing, thus, the formation of hydrogen bond between resistant β-tubulin and diethofencarb as mentioned above.

This model is consistent with the CD and fluorescence quenching results, in which carbendazim binding to resistant β-tubulin was not detected unless unusually high concentrations of the fungicide were used. Furthermore, the binding site identified by molecular docking would be located close to the GTP binding site, as indicated by superposition of the mammalian and *P. xanthii* β-tubulin models, in which the GTP-binding site and the proposed MBC-binding site nearly superpose (Fig. 5E). Moreover, residue 198 involved in fungal resistance does not contribute to MBC binding because this residue lies inside of the protein (Fig. 5F) and, therefore, is inaccessible to the fungicide. Thus, we can conclude that the binding of MBC fungicides to Ser138 and Thr178 may lead to a conformational change of β-tubulin; that results in its inactivation and consequently the inhibition of fungal growth, whereas the E198A mutation alters the topology of this binding site, rendering the protein resistant to MBC fungicides.

To confirm the role of Ser138 and Thr178 in the binding of MBC fungicides to β-tubulin, we performed site-directed mutagenesis to substitute these residues with alanine and generated the S138A and T178A mutant proteins. The mutant β-tubulins were expressed in *E. coli* and purified to homogeneity. Following purification, sensitive, resistant and mutant β-tubulins were subjected to SDS-PAGE analysis (Figure S7). The effect of carbendazim on the secondary structure of the mutant proteins was evaluated using circular dichroism (Figure S8). As observed for sensitive β-tubulin, progressive changes in the CD spectra of the S138A mutant protein were observed with increasing concentrations of carbendazim. In contrast, no variations in the CD spectra of the T178A mutant were observed. However, both mutants exhibited abnormal CD spectra in the absence of fungicide, becoming more pronounced for the T178A mutant, in which a characteristic CD spectrum was not observed. These findings led us to hypothesize a possible misfolding of the mutant proteins under native conditions.

## Discussion

The single-site nature of the mode of inhibition of MBC fungicides and previous misuse has led to the undesirable development of resistance in target pathogens, eventually reducing their efficacy and utility^28,29^. The unique target of MBC fungicides is the β-tubulin subunit of tubulin. Therefore, resistance to these fungicides in phytopathogenic fungi has rapidly evolved through the selection of mutants in the target gene such as the typical mutations E198A or F200Y^30^. As previously described in *Venturia nashicola*, causal agent of pear scab, and grey mold *Botrytis cinerea*, was described that point mutations in β-tubulin genes reduced carbendazim biding to tubulin-like proteins^31^. However, the mechanism by which those mutations affect fungicide binding is unknown.

Studies on the mechanism of action of MBC fungicides are essential to understand the interaction between the fungicide and the target protein. Recently, Zhou *et al*.^32^ suggested the binding site of MBC fungicides in the interface. However, in the present study we aimed to go further deciphering the precisely the structural base of binding site and the bases of MBC-resistance to aid in the development of novel, useful and long-term control strategies. We have previously reported that resistance to MBC fungicides in the cucurbit powdery mildew pathogen *P. xanthii* is based on the E198A mutation^13^. In this study, we have characterized the basis for this resistance using carbendazim as a representative member of this fungicide family. We provided biochemical and biophysical evidence of how the E198A mutation in β-tubulin promotes subtle conformational changes in β-tubulin that confer resistance to the fungicide. We hypothesized that carbendazim may inhibit β-tubulin by inducing conformational changes. Two lines of evidence supported this suggestion. First, the addition of subinhibitory concentrations of carbendazim to β-tubulin induced changes in the far-UV CD spectra, indicating a possible interaction that alters the secondary structure of the protein, as previously described^26,33^. We excluded a possible natural denaturation of the protein based on two observations: (*i*) in the sensitive β-tubulin, the α-helical content gradually decreased with corresponding enrichment in β-sheet content with increasing concentrations of carbendazim, and (*ii*) the secondary structure of the resistant β-tubulin remained invariable in the presence of carbendazim.

Notably, the highest concentration permitted for phytotherapy (3.3 mM) resulted in a dramatic change in the secondary structure. The presence of four disulphide bonds in β-tubulin led us to propose that carbendazim induces the reduction of the disulphide bonds present in β-tubulin as an additive effect that contributes to the instability of the β-tubulin dimers and, consequently, the failure in the assembly of final microtubules. According to the conformational changes in sensitive β-tubulin induced by the binding of carbendazim, we also detected a decrease in GTPase activity compared with that of resistant β-tubulin. Because CD analysis is not suitable for studies on the molecular details of protein structure, fluorescence techniques must be alternatively used to decipher small changes at the molecular level^26^. Fluorescence quenching provides various thermodynamic parameters, which allowed us to characterize the protein-ligand interaction in terms of affinity or spontaneity. Consistent with our hypothesis of conformational changes induced by the interaction between carbendazim and β-tubulin, we observed a differential behaviour between the sensitive and resistant β-tubulins in response to increasing concentrations of carbendazim. The thermodynamic parameters clearly indicated that carbendazim tightly binds to sensitive β-tubulin. First, upon the addition of carbendazim, a high value for *K*_*SV*_ was observed, a finding that has been previously reported and is related to major changes in the environment of the tryptophan residues induced by inhibitor binding^34^. Second, the negative values of free energy (Δ*G*) support the assertion that the binding process is highly spontaneous^35^. The results obtained for resistant β-tubulin reflected that carbendazim could bind to the protein, albeit with lower affinity and less spontaneously than the sensitive β-tubulin because *K*_*SV*_ and *K* were lower and Δ*G* was less negative than those observed for sensitive β-tubulin. We initially anticipated a contradiction with our model proposed by CD analysis; however, notably, the concentrations of carbendazim required to induce variations in the fluorescence quenching was far greater than the concentration used in all our analyses (3.3 mM), which is the concentration authorized for use on crops. Thus, it is unlikely that this interaction occurs under natural circumstances, or even that lethality of the fungicide is due to the target β-tubulin or an additional and nonspecific mechanism. We propose that the spontaneous binding of carbendazim to sensitive *Podosphaera xanthii* β-tubulin modifies its conformation, affecting proper polymerization into microtubules, which does not occur for resistant β-tubulin due to the conformational change induced by the E198A mutation.

These findings lead to the question of the localization of binding site for carbendazim and other MBCs in the β-tubulin subunit. Studies on competitive inhibition of certain MBCs from the benzimidazole chemical class, such as mebendazole, oxibendazole and fenbendazole, and the microtubule polymerization inhibitor colchicine, have demonstrated that these inhibitors bind to the intermediate domain between α- and β-tubulin, which is known as the colchicine-binding site^36^. This site has also been previously suggested as a common benzimidazole binding site^37^. Thus, it could be assumed that other MBCs, which induce similar modifications in microtubule dynamics, would also target this binding site. However, it has also been proposed that benomyl, which also belongs to the benzimidazole chemical class, does not compete with colchicine, and thus, an alternative specific binding site must be present in β-tubulin^38^. Carbendazim is chemically similar to benomyl and both possess more complex chemical structures than competitive inhibitors of colchicine; therefore, it is tentative to speculate that both MBC fungicides bind to a site that differs from the colchicine binding site. Based on our docking studies, we propose a binding site for MBCs that most likely involves the residues Ser138 and Thr178. This MBC binding site was identified through molecular docking using two chemically different fungicides, carbendazim, as a representative of benzimidazole chemical group, and diethofencarb, as a member of N-phenyl carbamates group. In case of carbendazim, Ser138 and Thr178 were found to tentatively bind the fungicide. As shown in Fig. 5, the proposed MBC binding site is physically close to the GTP binding domain^24^; it does not include residues 198 and 200 that are typically involved in resistance; and it is distinct from the colchicine binding site (Cys239, Cys345)^39^, which is located on the opposite side of the protein. In case of diethofencarb, only binding to resistant β-tubulin was predicted by *in silico* analyses performed in this study. In fact, those analyses suggested the existence of a negative cross-resistance phenomenon between carbendazim and diethofencarb. This fact has been previously described in *Botrytis cinerea* and *Neurospora crassa* where MBC resistance isolates showed enhanced sensitivity to diethofencarb^7,17,40^. This negative cross-resistance provides an extraordinary method to combat resistance by mean of benzimidazole-phenylcarbamate mixture, which has been used commercially as an anti-resistance strategy against *Botrytis cinerea* in several crops since late 1990s in China^40^. The binding pocket proposed in the present study could explain why double-resistant mutants frequency is often low since residues that participate in binding change their orientation due to the substitution E198A.

The computational model allowed us to also visualize the structural bases of MBC resistance. In the resistant β-tubulin, the E198A mutation induced a structural rearrangement of the entire protein, affecting the proposed MBC binding site, in which Thr178 is twisted out, precluding the establishment of hydrogen bonds between the MBC fungicides and protein, thereby conferring resistance to MBC.

To evaluate the hypothesis that mutations in residues involved in MBC binding confer resistance to these fungicides, these residues were mutated to alanine and the resultant mutant proteins were analysed for protein folding and fungicide binding. The results indicated that the T178A mutant was unable to bind the fungicide; however, both S138A and T178A mutant proteins underwent dramatic misfolding in the absence of the fungicide, indicating the instability of these mutant proteins, therefore no further analyses were carried out with them.

## Methods

### Fungal isolates, plant material and fungicides

The P. xanthii isolates 2086 and SF60, which are sensitive and resistant, respectively, to MBC fungicides, were grown on cotyledons of zucchini (Cucurbita pepo) cv. Negro Belleza (Semillas Fitó) and maintained *in vitro* as previously described^41^. Plants were grown from seeds in a growth chamber at 25 °C under a 16 h photoperiod. Single-spore isolates were stored at −80 °C until analysis^42^. Non-commercial and technical grade formulations of the MBC fungicides carbendazim (Maypon Flow^®^, Agrodan S.A., Spain) and benomyl (Sigma-Aldrich) were used. A fungicide stock dispersion of carbendazim was prepared in water at 10 mg ml^−1^ as described previously for commercial QoI fungicides by Fernández-Ortuño *et al*.^11^; a water-soluble formulation was chosen to avoid protein denaturation due to organic solvent. Maypon Flow^®^ is composed by carbendazim 50%, therefore, calculations were performed to adjust concentrations as required for subsequent experiments. Benomyl stock solution was prepared in acetone at 1 mg ml^−1^. Stock solutions were diluted in sterile deionized water to yield solutions with the desired final concentrations.

### Heterologous expression of *P. xanthii* β-tubulins in yeast

The full-length coding sequence of the *P. xanthii TUB*2 gene (Genbank accession no. KC333362) was amplified from cDNA libraries of the *P. xanthii* isolates 2086 and SF60 using the primers pREP-TUB2F and pREP-TUB2R (Table S2), which incorporate *Xho*I and *Sma*I restriction sites, respectively, to direct the cloning in the yeast expression vector pREP-3X^43^. After digestion, the fragments were cloned into the vector to generate the pREP-E198 or pREP-A198 expression plasmids, containing the β-tubulin alleles that are responsible for sensitivity (E198) or resistance (A198), respectively, to MBCs. Inserts were sequenced to ensure the integrity of the sequences and plasmids were then transformed into *Schizosaccharomyces pombe* strain RA4134 (*h*-, *ale*6-210, *arg*3D, *his*3D, *leu*1-32, *ura*4DS/E) using the lithium acetate procedure^44^. Selection of transformants was performed on PMG minimal medium lacking leucine (auxotrophic selection) and supplemented with thiamine to prevent possible toxic effects of plasmid expression.

### Fungicide sensitivity assay

The sensitivity of *S. pombe* to fungicides was evaluated as previously described by Cools *et al*.^45^. Single colonies of *S. pombe*::PfTUB2-E198, *S. pombe*::PfTUB2-A198 and *S. pombe*::pREP-3X were resuspended in 200 μl of PBS on a microtiter plate. A 10-fold serial dilution of this culture was prepared, and 5 μl of each dilution was spotted onto PMG medium plates lacking thiamine, without fungicide or amended with the MBC fungicides carbendazim (3.3 mM) or benomyl (0.1 mM). After 5 days of incubation at 30 °C, the plates were scanned.

### *In vitro* expression and purification of *P. xanthii* α- and β-tubulins, immunoblot and mass spectrometry analyses of *in vitro* expressed proteins

To express the *P. xanthii* tubulin genes in *E. coli*, cDNAs of the genes encoding *TUB1* (α-tubulin) (GenBank accession number: KF767691) and *TUB2* (β-tubulin) were amplified and cloned into the inducible expression plasmids pET-29b (Novagen) and pDEST17 (Invitrogen), respectively. To determine the transcription initiation and termination sites of the *TUB1* gene, total RNA was used to generate 5′ and 3′ RACE (Rapid Amplification of cDNA Ends) products using the SMARTer^™^ RACE cDNA Amplification Kit (Clontech) as previously done for the *TUB2* gene^13^. The *P. xanthii TUB1* gene was amplified from this adapter-ligated cDNA library using the primers alphatubF and alphatubR (Table S2); and the Advantage 2 Polymerase Mix (Clontech).

Subsequently, the primers *tub*1p29F and *tub*1p29R (Table S2) were used to amplify the entire *TUB1* coding sequence and incorporate *Nde*I and *Not*I restriction sites. The *TUB1* coding sequence was digested and cloned into the pET-29b plasmid, which incorporates a six His-tag at the C-terminus of the protein. The primer pair *tub*2-207F and *tub*2-207R (Table S2) was used to amplify *PfTUB*2 coding sequence mentioned above, amplified fragments were cloned into the entry vector pDONR207 and the destination vector pDEST17, which incorporates a six His-tag at the N-terminus of the proteins, using the Gateway^®^ cloning technology (Invitrogen). The resulting the plasmids were designated pDEST17-E198 and pDEST17-A198, which encode MBC-sensitive and MBC-resistant β-tubulin proteins, respectively.

Site-directed mutagenesis was performed using the QuikChange Lightning Multi Site-Directed Mutagenesis Kit (Agilent Technologies) according to the manufacturer’s protocol. The template for mutagenesis encompassed the entire locus of the *TUB2* gene from the MBC-sensitive *P. xanthii* 2086 isolate cloned into pDEST17. Residues Ser138 and Thr178 were replaced by Ala using the primer pairs SDM138-F/-R and SDM178-F/-R, respectively (Table S2), generating the pDEST17-A138 and pDEST17-A178 plasmids.

For α-tubulin expression, the plasmid pET29b-TUB1 was transformed into competent *E. coli* BL21 (DE3) cells using heat shock, and protein expression was induced with 1 mM IPTG (isopropyl-β-D-thiogalactopyranoside) when the optical density at 600 nm (OD_600 nm_) reached 0.7. Cells were incubated at 37 °C for 6 h and harvested by centrifugation. For β-tubulin expression, the plasmids pDEST17-E198, pDEST17-A198, pDEST17-A138 and pDEST17-A178 containing the MBC-sensitive, MBC-resistant and site-directed β-tubulin mutant sequences, respectively, were transformed into chemically competent *E. coli* BL21-AI cells (Invitrogen) using heat shock. Protein expression was induced with the addition of 10 mM arabinose (Sigma-Aldrich) at an OD_600 nm_ of 0.4. Cells were incubated at 18 °C for 12 h and harvested by centrifugation. All cell pellets were frozen in liquid nitrogen and stored at −80 °C overnight to increase the yield of protein recovery.

For protein purification, cell pellets were thawed, resuspended in lysis buffer (1 × CellLytic^TM^ MT Buffer [Sigma-Aldrich]) supplemented with 1 mM of protease inhibitor Phenylmethylsulfonyl fluoride (PMSF) and incubated at room temperature for 1 h; the remaining cells were disrupted by sonication on ice using a UP100H sonicator (Hielscher). The crude lysate was clarified by centrifugation at 8000 g for 10 min prior to mixing with 5 ml of equilibrated HIS-Select^®^ Nickel Affinity Gel (Sigma-Aldrich) and incubated for 2 h at room temperature.

After extensive washes using washing buffer (50 mM Na_3_PO_4_ [pH 8], 0.5 M NaCl and 10 mM imidazole), proteins were eluted with 10 ml of elution buffer (50 mM Na_3_PO_4_ [pH 8], 0.3 M NaCl and 500 mM imidazole). Proteins were dialyzed against 20 mM Tris-HCl (pH 8), 50 mM NaCl, concentrated by ultrafiltration using Pierce^®^ Concentrators 20 K MWCO (Thermo Scientific) and stored at −20 °C until analysis.

Prior to further analysis, expressed proteins were characterized by Western blot and mass spectrometry analyses. For immunoblot analysis, purified proteins were electrophoresed on 12% SDS-PAGE gels and electrotransferred onto polyvinylidene difluoride (PVDF) membranes using the Trans-Blot Turbo electrophoretic transfer cell (Bio-Rad). Blots were probed with a 1:1000 dilution of a rabbit monoclonal anti-His-tag antibody (Rockland). The membranes were then incubated with a 1:20,000 dilution of a horseradish peroxidase-conjugated anti-rabbit antibody (Bio-Rad), and bands were visualized by chemiluminescent detection using an ECL Western blotting analysis system (Thermo Scientific).

The identity and integrity of the proteins were confirmed using MALDI-TOF/TOF analysis of the peptides released following enzymatic digestion using trypsin^46,47^. Briefly, digestion was performed using 10 ng ml^−1^ of trypsin and 50 mM ammonium bicarbonate and overnight incubation at 37 °C. Following digestion, the resultant peptides were extracted three times with 20 ml of 5% trifluoroacetic acid in 50% acetonitrile and concentrated to 5 ml in this solvent. All analyses were performed using a 4700 MALDI-TOF/TOF Proteomics Analyzer (AB SCIEX). Peptide mixtures were analyzed using a saturated solution of α-cyano-4-hydroxycinnamic acid in 50% acetonitrile/0.1% trifluoroacetic acid. For database searches, no restrictions were placed on the species or organism. Spectra were calibrated using matrix and tryptic autodigestion ion peaks as internal standards.

### Circular dichroism spectroscopy

The secondary structure of the proteins following fungicide binding was determined using circular dichroism (CD) spectroscopy^26^. MBC-sensitive and MBC-resistant β-tubulin proteins (1 mM), alone or in combination with carbendazim (0.5, 1.5 or 3.3 mM) were incubated in PEM buffer^48^ for 30 min at room temperature. CD spectra were recorded using multiple scans at 25 °C in a JASCO J-815 spectropolarimeter equipped with a JASCO PTC-423S Peltier temperature control system (JASCO). Far-UV CD spectra were recorded over the wavelength range of 190–250 nm with a scan speed of 200 nm min^−1^ and a response time of 1 s, using a 0.3-cm path-length cuvette. The results were expressed as mean residue ellipticity as described elsewhere^26^. Secondary structure estimation from CD spectra was obtained using K2D software (www.ogic.ca/projects/k2d3/).

### Fluorescence quenching

To estimate the thermodynamics of carbendazim binding to β-tubulin, fluorescence quenching was used^49^. The intrinsic fluorescence of β-tubulin tryptophan residues (W21, W101, W344, W397) in absence or in combination with various concentrations of carbendazim (0.5, 1.5, 2.5, 3.3, 5.2, 15 or 25 mM) was monitored in a PTI QM-2000–6 spectrofluorometer (Photon Technology International) using a 0.3-cm pathlength cuvette with an excitation wavelength of 295 nm and an emission range from 300–400 nm. The fluorescence quenching data were analysed according to the Stern-Volmer equations^26^. This way, parameters such as the Stern-Volmer quenching constant (*K*_*SV*_), the binding constant (*K*), the number of binding sites (*n*) and the free energy of binding (*∆G*^0^_binding_) were estimated.

### Protein modeling and molecular docking

The SWISS-MODEL workspace (swissmodel.expasy.org/workspace/) was used to perform automated protein structure homology modelling of *P. xanthii* β-tubulins using the crystal structure of *Bos taurus* β-tubulin (PDB: 1JFF) as the template. To identify potential binding sites and the binding affinities of carbendazim (PubChem ID: 25429) and diethofencarb (PubChem ID: 91742) for β-tubulin, automated molecular docking was performed using the web-based SwissDock program (www.swissdock.ch/docking). The docking was performed using the ‘Accurate’ parameter at otherwise default parameters, with no region of interest defined (blind docking). Modeling and docking results were visualized using UCSF Chimera v1.12 software.

## Electronic supplementary material

Supplementary information

video
