# Supplementary material for: Analysis of β-tubulin-carbendazim interaction reveals that binding site for MBC fungicides does not include residues involved in fungicide resistance
Source: Sci Rep. 2018 May 8;8:7161. doi: 10.1038/s41598-018-25336-5 (PMC5940828; doi:10.1038/s41598-018-25336-5)
Supplement: Supplementary file 1 — Supplementary information [file 41598_2018_25336_MOESM1_ESM.docx]

**Supplementary Information**

**Analysis of β-tubulin-carbendazim interaction reveals that binding site for MBC fungicides does not include residues involved in fungicide resistance**

**Authors**

David Vela-Corcía, Diego Romero*, Antonio de Vicente and Alejandro Pérez-García

**Affiliation**

*Instituto de Hortofruticultura Subtropical y Mediterránea ‘‘La Mayora’’ - Universidad*

*de Málaga - Consejo Superior de Investigaciones Científicas (IHSM-UMA-CSIC),*

*Departamento de Microbiología, Universidad de Málaga, Bulevar Louis Pasteur 31*

*(Campus Universitario de teatinos), 29071 Málaga, Spain*

* diego_romero@uma.es

**Supplementary figures**

-Fig S1. In vitro expression and purification of α-tubulin and β-tubulin subunits from *P. xanthii*.

-Fig S2. Circular dichroism analysis of *P. xanthii* MBC-sensitive and MBC-resistant β-tubulin proteins.

-Fig S3. Circular dichroism analysis of carbendazim.

-Fig S4. Effect of carbendazim on the secondary structures of *P. xanthii* α-tubulin and β-tubulins.

-Fig S5. Variations in the percentages of secondary structures of *P. xanthii* β-tubulins in response to increasing concentrations of carbendazim.

-Fig S6. Fluorescence emission spectrum of carbendazim.

-Fig S7. In vitro expression and purification of *P. xanthii* sensitive, resistant and mutant β-tubulins.

-Fig S8. Circular dichroism (CD) analysis of the effect of carbendazim on the secondary structures of *P. xanthii* β-tubulin mutants S138A and T178A.

**Supplementary data**

Movie S1. Animation of the conformational change caused by the E198A mutation in the *P. xanthii* β-tubulin.

Data S1. Mass spectrometry analysis of α-tubulin and β-tubulin.

**Supplementary Tables**

Table S1. Percentage of secondary structure of *P. xanthii* β-tubulins estimated from CD spectrum data.

Table S2. Primers used in this study.

**Figure S1**


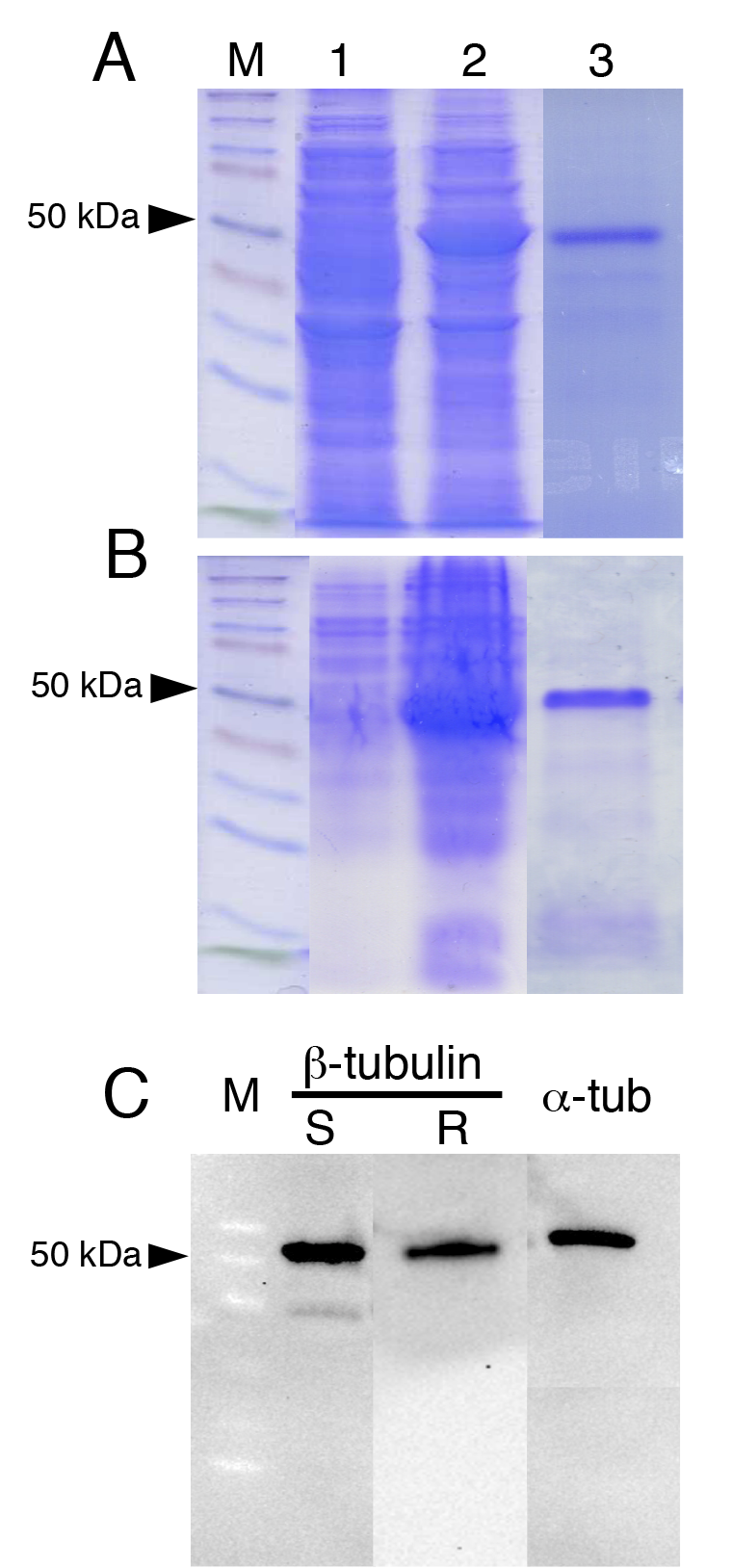


**Figure S1**. In vitro expression and purification of α-tubulin and β-tubulin subunits from *P. xanthii*. (A and B) SDS-PAGE gels under reducing conditions of α-tubulin (A) and MBC-sensitive β-tubulin (B) at various steps in the purification. Lanes: 1, total cellular protein without induction; 2, total cellular protein after induction with 1 mM IPTG for α-tubulin expression (A) or 10 mM arabinose for MBC-sensitive β‑tubulin expression (B); 3, imidazole elution of the Ni^+2^-charged resin containing the corresponding protein. The numbers on the left indicate the molecular weights of the proteins in kDa. (C) Immunoblot analysis using specific monoclonal anti-His antibodies (1:1,000) indicating the homogeneity of purified MBC-sensitive (S) and MBC-resistant (R) β-tubulin and α-tubulin proteins. M, Spectra Multicolor Broad Range Protein Ladder (Thermo Fisher Scientific, MA, USA). Gels were cropped to avoid intermediary protein purification steps, showing only final protein elution.

**Figure S2**


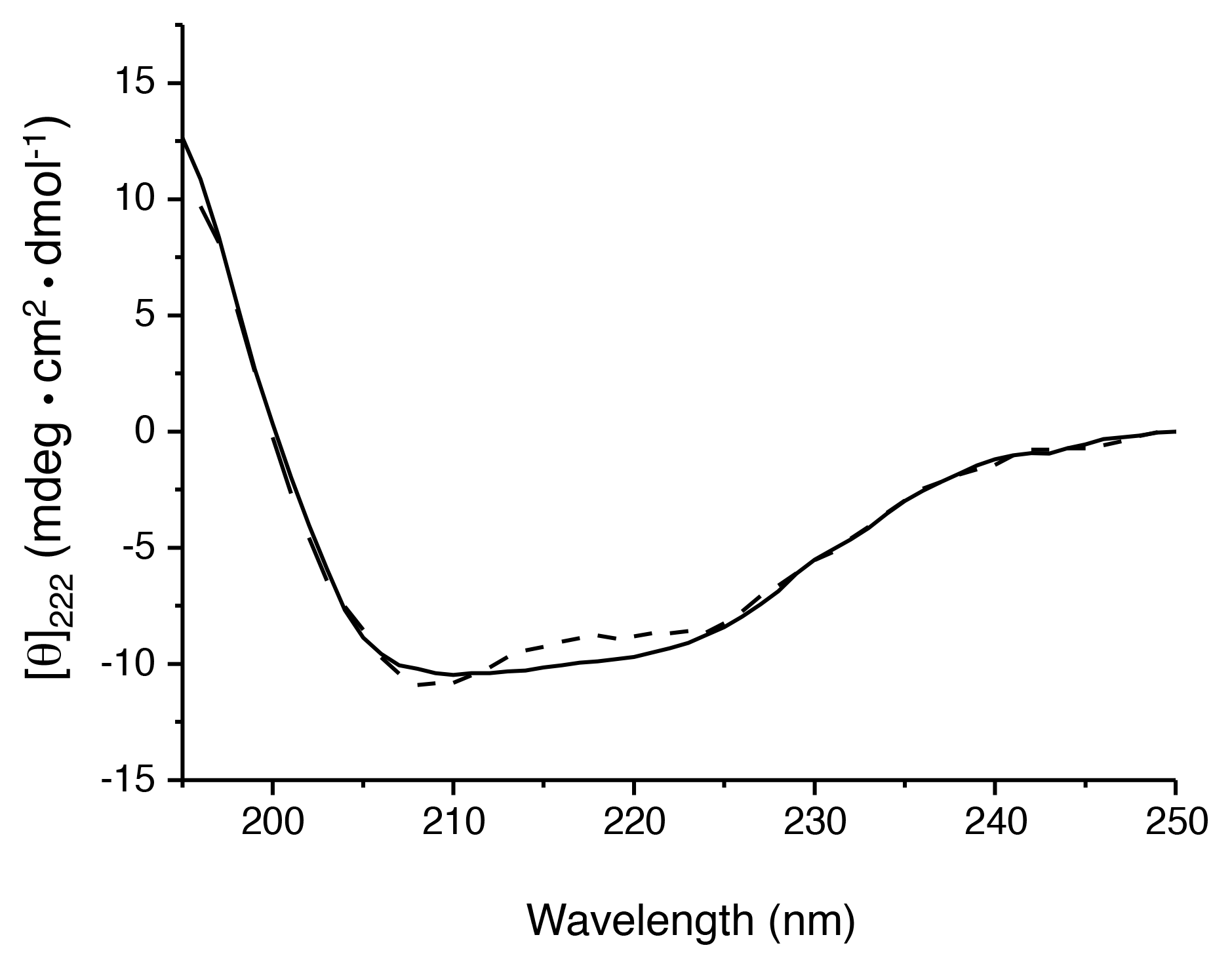


**Figure S2**. Circular dichroism analysis of *P. xanthii* MBC-sensitive and MBC-resistant β-tubulin proteins. The circular dichroism spectrum of MBC-sensitive β-tubulin (solid line) slightly differs from that of the MBC-resistant β-tubulin (dashed line), indicating variations in the secondary structure content resulting from the E198A mutation. Each spectrum represents the average of three scans.

**Figure S3**

A

B

**Figure S3**. Circular dichroism analysis of carbendazim. A) Circular dichroism spectrum of carbendazim 3.3mM. B) Superposition of β-tubulin circular dichroism (dashed line) and theoretical spectrum from sum of carbendazim 3.3mM and β-tubulin spectra (red line).

**Figure S4**

**
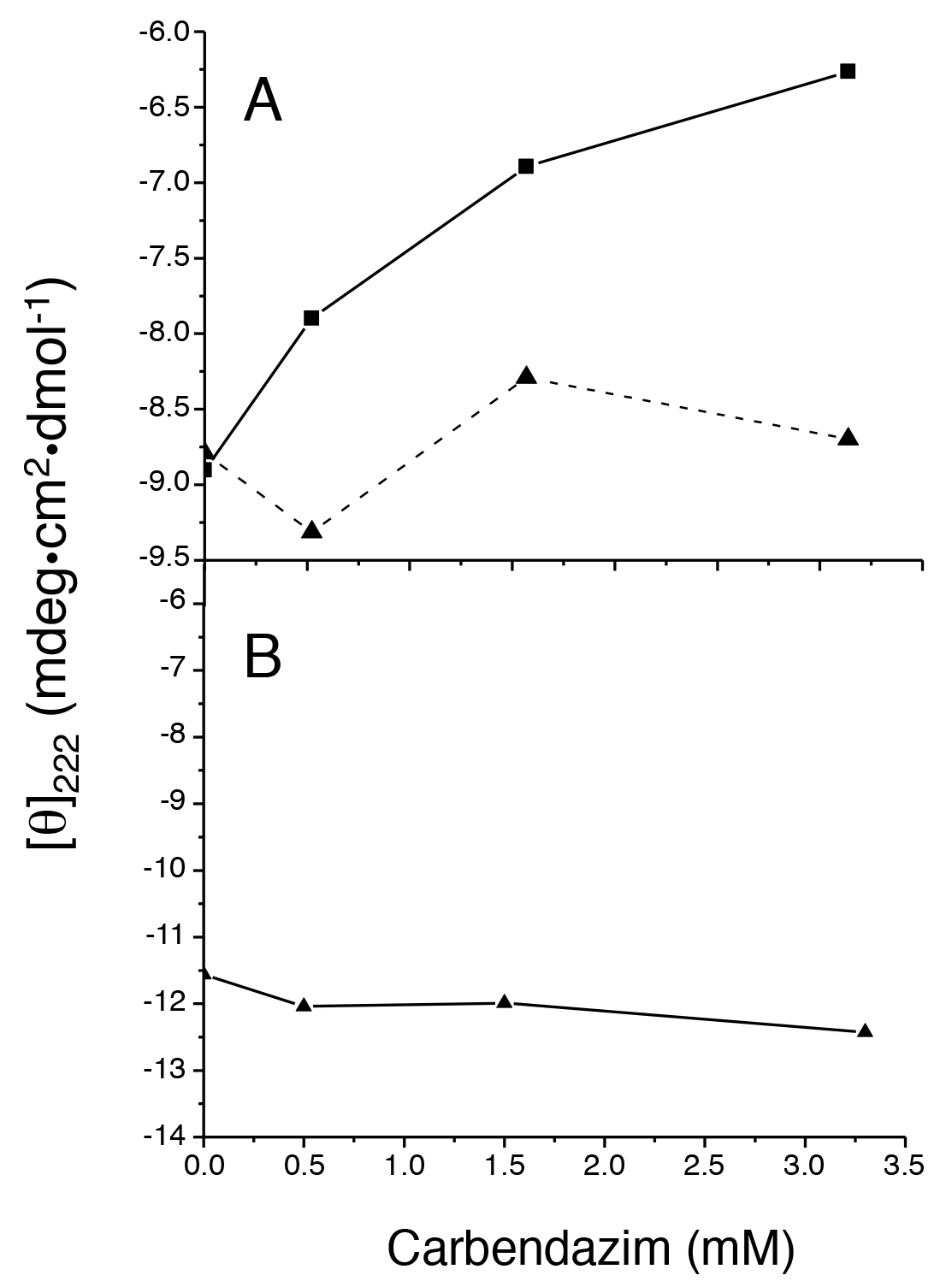
**

**Figure S4**. Effect of carbendazim on the secondary structures of *P. xanthii* α-tubulin and β-tubulins. (A) The mean residue ellipticity at 222 nm of MBC-sensitive β-tubulin (solid line) changes with increasing concentrations of carbendazim, whereas MBC-resistant β-tubulin (dashed line) remains invariable. (B) The mean residue ellipticity at 222 nm of α-tubulin, which is not a known target of MBC fungicides, did not change with increasing concentrations of carbendazim.

**Figure S5**


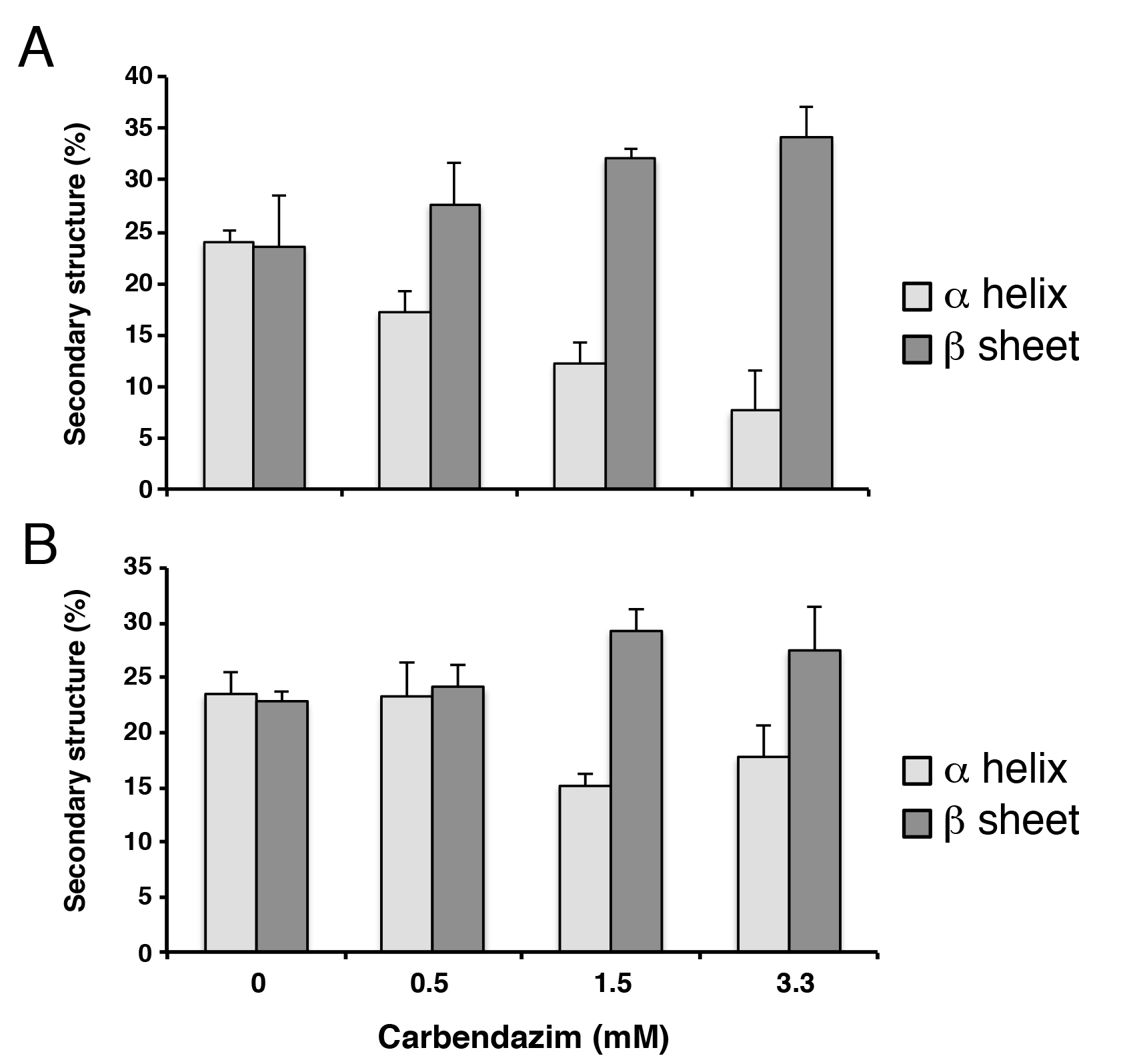


**Figure S5**. Variations in the percentages of secondary structures of *P. xanthii* β-tubulins in response to increasing concentrations of carbendazim. (A) For MBC-sensitive β-tubulin, a progressive decrease in the α-helical content and a progressive increase in the percentage of β-sheet were observed in the range of assayed carbendazim concentrations. (B) For MBC-resistant β-tubulin, only an approximately 5% decrease in the α-helical content and an approximately 5% increase in the percentage of β-sheet were observed.

**Figure S6**

**Figure S6**. Fluorescence emission spectrum of carbendazim 3.3mM. Carbendazim emission maximum was 335nm, which is different to the maximum observed for β‑tubulin.

**Figure S7**


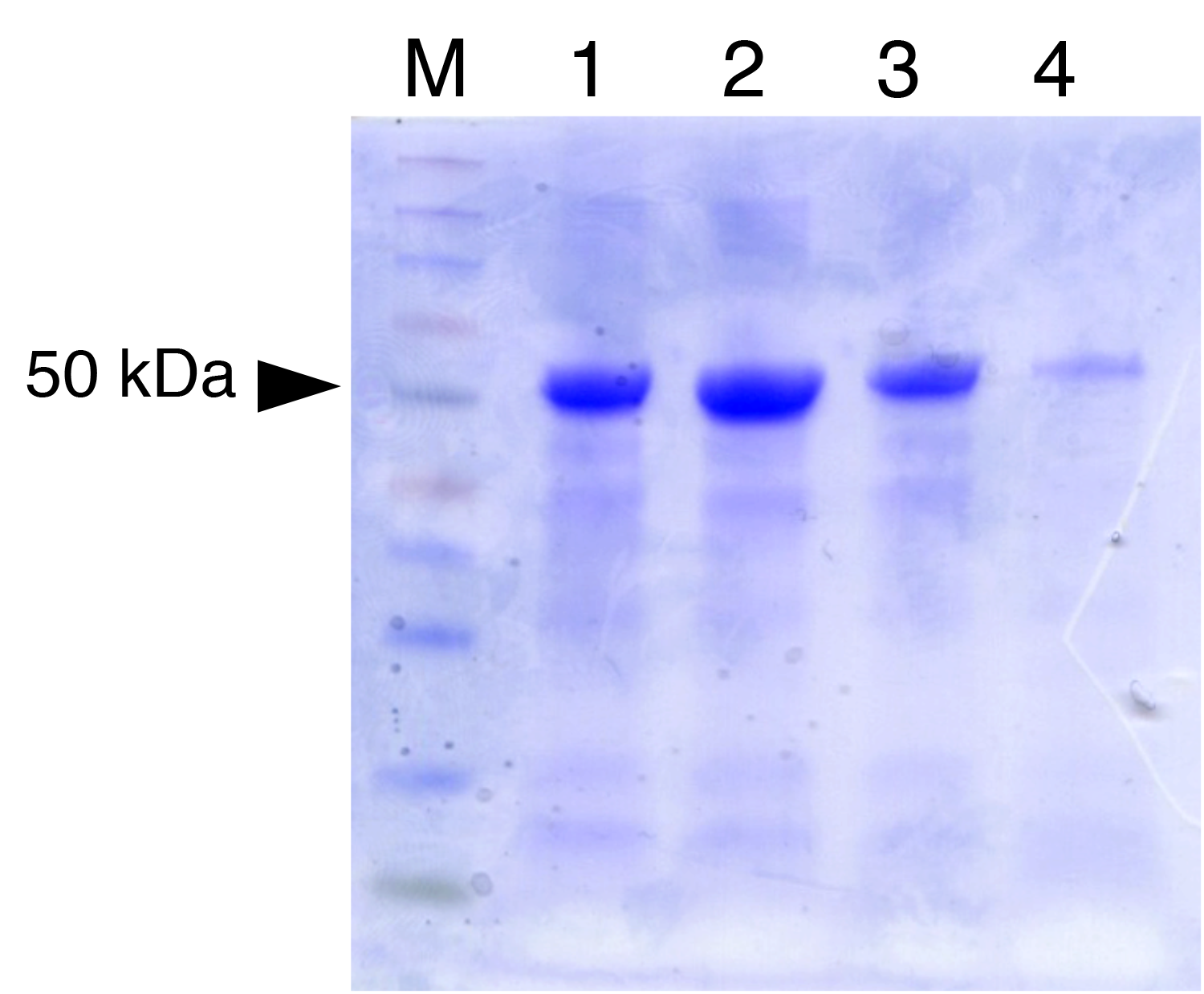


**Figure S7**. In vitro expression and purification of *P. xanthii* sensitive, resistant and mutant β-tubulins. SDS-PAGE analysis under reducing conditions of MBC-sensitive MBC-resistant and mutant β-tubulins. Lanes indicate imidazole elutions from a Ni^+2^-charged resin for MBC-sensitive β-tubulin (1), MBC-resistant β-tubulin (2) and β-tubulin mutants S138A (3) and T178A (4). M, Spectra Multicolor Broad Range Protein Ladder (Thermo Fisher Scientific). The numbers on the left indicate the molecular weights of the proteins in KDa.

**Figure S8**


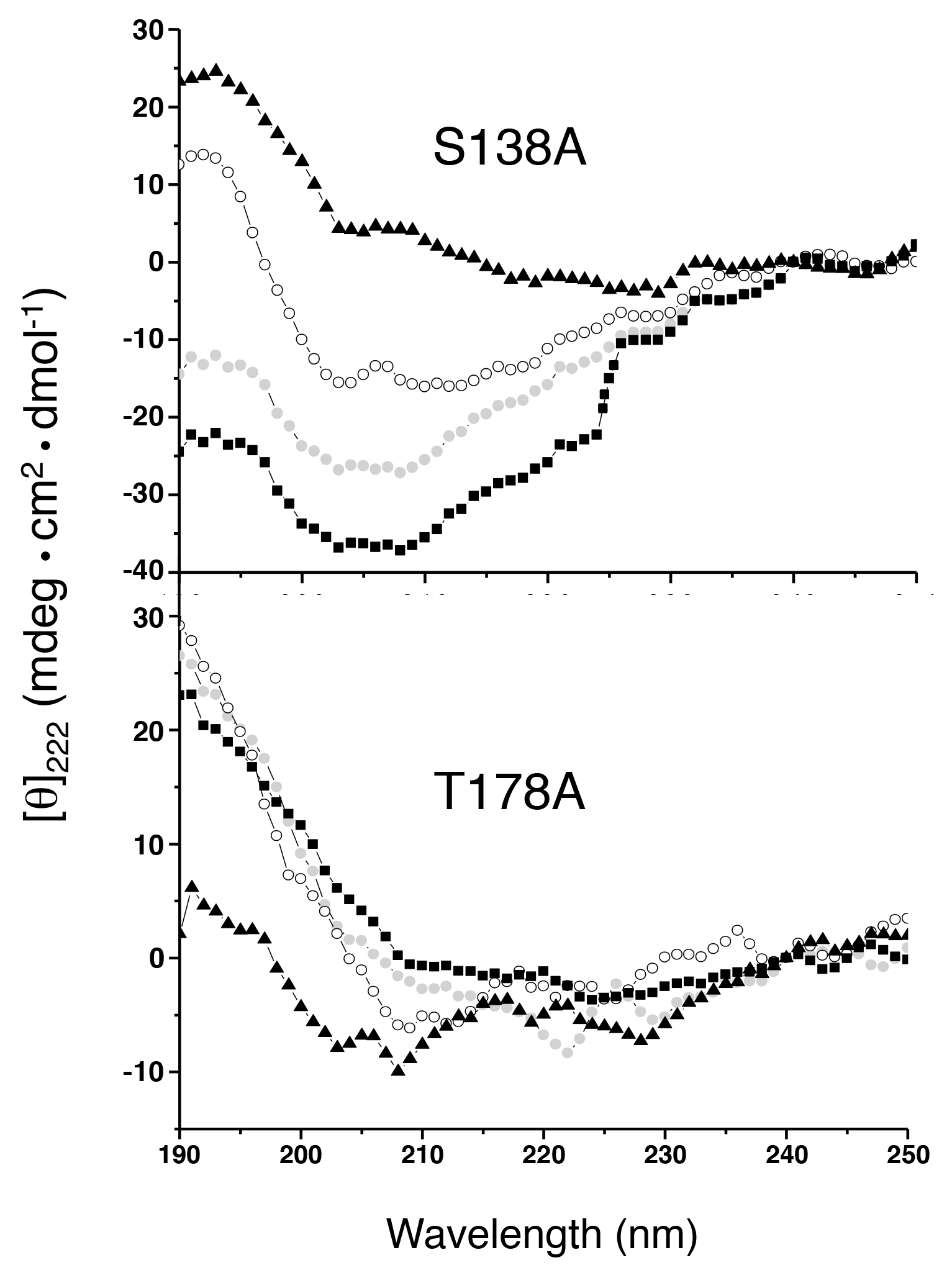


**Figure S8**. Circular dichroism (CD) analysis of the effect of carbendazim on the secondary structures of *P. xanthii* β-tubulin mutants S138A and T178A. CD spectra of β-tubulin mutants S138A and T178A in the absence (■) and presence of 0.5 (⏺), 1.5 (○) and 3.3 mM (▲) carbendazim. In the presence of carbendazim the CD spectra of mutant S138A were progressively altered indicating significant changes in the secondary structure of the protein. Identical carbendazim concentrations were used for the T178A mutant, and no significant changes were observed. Notably, both proteins exhibited non-canonical spectra in the absence of fungicide, indicative of misfolded of proteins.

| **Table S1**. Percentage of secondary structure of *P. xanthii* β-tubulins estimated from CD spectrum data | | |
| --- | --- | --- |
| **Secondary structure** | **Sensitive (E198)** | **Resistant (A198)** |
| α-helix | 22.17% | 22.38% |
| β-sheet | 25.06% | 24.61% |

| **Table S2**. Primers used in this study | | |
| --- | --- | --- |
| **Primer Name** | **Sequence (5´- 3´)** | **Product size (bp)** |
| pREP-TUB2F | AAAACTCGAGATGCGTGAAATTGTACACATCTT | 1,480 |
| pREP-TUB2R | AAAACCCGGGTTATTCTTCCGGTTGCATGGGTG |  |
| alphatubF | AGTTCGCACTGGCACCTACC | 400 |
| alphatubR | GGGCGTATGCAACAAGAGGG |  |
| *tub*1p29F | AAAACATATGAGGGAAGTCATAAGCATCAAC | 1,500 |
| *tub*1p29R | AAAAGCGGCCGCATATTCTGCTTCGCCACCCTCG |  |
| *tub*2-207F | AAAAAGCAGGCTCTATGCGTGAAATTGTTC | 1,600 |
| *tub*2*-*207R | AGAAAGCTGGGTTTATTCTTCCGGTTGCATG |  |
| SDM138-F | GGATTTCAAATAACACATGCGCTTGG | 6,354 |
| SDM138-R | GTCCCTCCTCCAAGCGCATGTG |  |
| SDM178-F | CTCCAAAGGTGTCTGATGCGGTTGTTGAG | 6,354 |
| SDM178-R | CATTGTATGGCTCAACAACCGCATCAGAC |  |


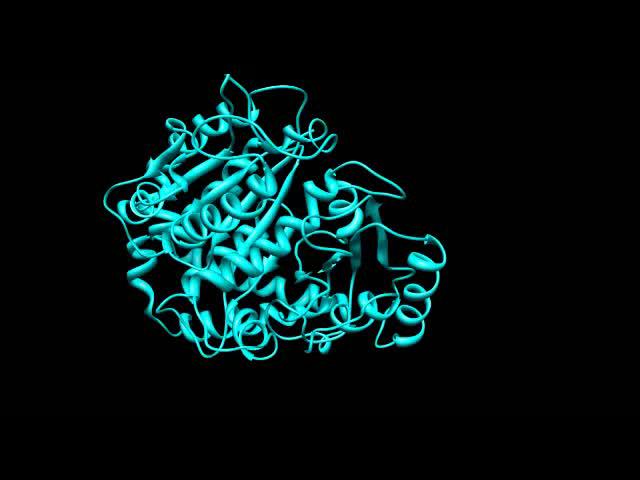
**Movie S1**

**Movie S1**. Animation of the conformational change caused by the E198A mutation in the *P. xanthii* β-tubulin. Observe how a single amino acid substitution triggers the rearrangement of the entire protein structure, which is responsible for the transition from a sensitive β-tubulin to a variant form that is resistant to MBC fungicides.

**Data S1**

Mass spcetrometry of α-tubulin.

Mass spcetrometry of β-tubulin.
